# Three functional mutation sites affect the immune response of pigs through altering the expression pattern and IgV domain of the CD4 protein

**DOI:** 10.1186/s12860-020-00333-7

**Published:** 2020-12-09

**Authors:** Weiya Zhang, Juan Ni, Jie Zhang, Lu Zhang, Huanhuan Zhou, Changzhi Zhao, Mengjin Zhu, Haiyan Wang, Jianlin Han, Xinyun Li, Shuhong Zhao

**Affiliations:** 1 Mountainous Area Research Institute of Hebei Province, Hebei Agricultural University, No. 289 Lingyusi Street, Lianchi District, Baoding, Hebei Province 071001 P. R. China; 2 Zhejiang Cancer Research Institute, Hangzhou, China; 3 Huazhong Agricultural University, No.1 Shizishan Street, Hongshan District, Wuhan, Hubei Province 430070 P. R. China; 4 Key Laboratory of Agricultural Animal Genetics, Breeding, and Reproduction of the Ministry of Education, Swine Genetics and Breeding of Ministry of Agriculture and Rural Affairs, Huazhong Agricultural University, No.1 Shizishan Street, Hongshan District, Wuhan, Hubei Province 430070 P. R. China; 5 Livestock Genetics Program, International Livestock Research Institute (ILRI), Nairobi, Kenya; 6 The Cooperative Innovation Center for Sustainable Pig Production, Wuhan, China

**Keywords:** Functional mutations, *CD4* gene, Translation, Membrane localization, Immune response, Pig

## Abstract

**Background:**

The CD4 protein is an important surface marker of T lymphocytes, which can mediate the antigen presentation process by interacting with MHC II and TCR molecules in human and mouse.

**Results:**

In this study, two haplotypes (A and B) of the *CD4* gene were found within Chinese indigenous and Western commercial pig breeds. These two haplotypes were defined by 22 fully linked SNPs in the CDS region of the *CD4* gene. The expression level and localization of the CD4 protein were significantly different between haplotypes A and B. Transcriptome analysis revealed that the immune response-related genes and signaling pathways were down-regulated in genotype AA. Finally, three linked functional SNPs were identified, which affected the expression level and membrane localization of the CD4 protein in pigs. These three SNPs led to the replacements of two amino acids in the IgV1 domain of the CD4 protein, and related to the function of the CD4 protein in the immune response.

**Conclusion:**

These three linked SNPs were the key functional mutation sites in the *CD4* gene, which played important roles in the immune response, and could be utilized as new molecular markers in breeding for disease resistance in pigs.

**Supplementary Information:**

The online version contains supplementary material available at 10.1186/s12860-020-00333-7.

## Background

The CD4 molecule belongs to a class of differentiation antigens expressed on the surface of immune response-related cell, such as T cells [1, 2]. T cells play a vital role in anti-pathogen infection, autoimmune disease, and antitumor immunity. Based on the expressions of the surface makers of CD4 and CD8, T cells possess four developmental stages. The first stage contains the most immature thymocytes with double negative (DN) CD4 and CD8. The second stage is characterized by up-regulation of both CD4 and CD8, producing double-positive (DP) thymocytes. The third stage contains CD8 or CD4 single-positive (SP) thymocytes via positive selection of MHC I or II molecules [3]. CD_4_^+^ T cells eliminate pathogens by helping innate immune responses, B cells, and CD_8_^+^ T cells. Moreover, cytotoxic CD4^+^ T cells (CD_4_^+^ CTLs) can directly induce the apoptosis of target cells that have overexpressed MHC II due to viral infection [4]. In addition, the *CD4* gene plays an important role in T cell development. In humans, the CD4 protein contains four Ig-like extracellular domains, one transmembrane domain, and a C-terminal cytoplasmic tail [5–7]. The expression level of the CD4 protein corresponds to cell lineages with different specific functions during T cell development. Therefore, the regulation of the CD4 protein level is linked to developing T cells.

Previous studies indicated that the expression level of the *CD4* gene was strictly controlled by five stage-specific cis-elements, which included silencer (S4), proximal enhancer (E4p), distal enhancer, thymocyte enhancer, and intronic enhancer. Among them, E4p was required to maintain a stable level of *CD4* gene expression during positive selection in DP thymocytes, S4 repressed the expression level of the *CD4* gene in DN and cytotoxic CD_8_^+^ T cells, and E4m promoted the expression level of the *CD4* gene in post-selected stages [8]. Moreover, five transcription factors regulated the expression level of the *CD4* gene by binding to cis-elements during T cell development, which included Runx1, Runx3, HEB, TCF1, and E2A [9]. In addition, the activity of T helper cells was reduced due to the production of Il-2 in *CD4* knockout mice [10].

CD4 can mediate the antigen presentation process by interacting with MHC II and the TCR signaling pathway. The inhibition of CD4–MHC II interaction weakened the immune response of T cells to exposed antigen, and the reduction in the expression level of the CD4 protein impaired signal transduction of the TCR pathway in T lymphocytes of mice [11]. Moreover, the ability to resist Leishmania infection was impaired in CD4 knockout mice [10].

Some mutations in the *CD4* gene are related to immune diseases or viral infection. In humans, three SNPs in the promoter region of the *CD4* gene were related to type 1 diabetes mellitus [12]. A trait-association study indicated the relationship of two SNPs in the enhancer regions to the severity of rheumatoid arthritis [13]. Furthermore, one C to T substitution at nucleotide position 868 of the *CD4* gene was related to HIV-1 acquisition and disease progression in Kenyans [14–16]. In macaques, one amino acid replacement at position 39 of the CD4 protein was responsible for restricting HIV infection [17]. Matsubara et al. found that there were two haplotypes (CD4.A&CD 4.b) in the full-length of CD4-CDS in Japanese miniature pigs, and CD4 protein encoded by these two haplotypes showed different binding capacities to CD4 antibodies due to the amino-acid substitutions [18]. Nevertheless, the functional mutation sites of the *CD4* gene in swine are largely unknown.

In this study, 22 fully linked SNPs in the CDS region of the *CD4* gene were identified in F2 population of Duroc × Erhualian, which led to the formation of two haplotypes. Moreover, the expression level, membrane localization, and the immune responses of the CD4 protein were different between different haplotypes. Finally, the key mutation sites that led to functional differences between two haplotypes were identified. This study offers an important marker for regulating the immune response of pigs, and this molecular marker can be used in breeding for disease resistance in pigs.

## Results

### Fully linked SNPs in the CDS region led to the formation of two haplotypes of the *CD4* gene

**Fig. 1:**
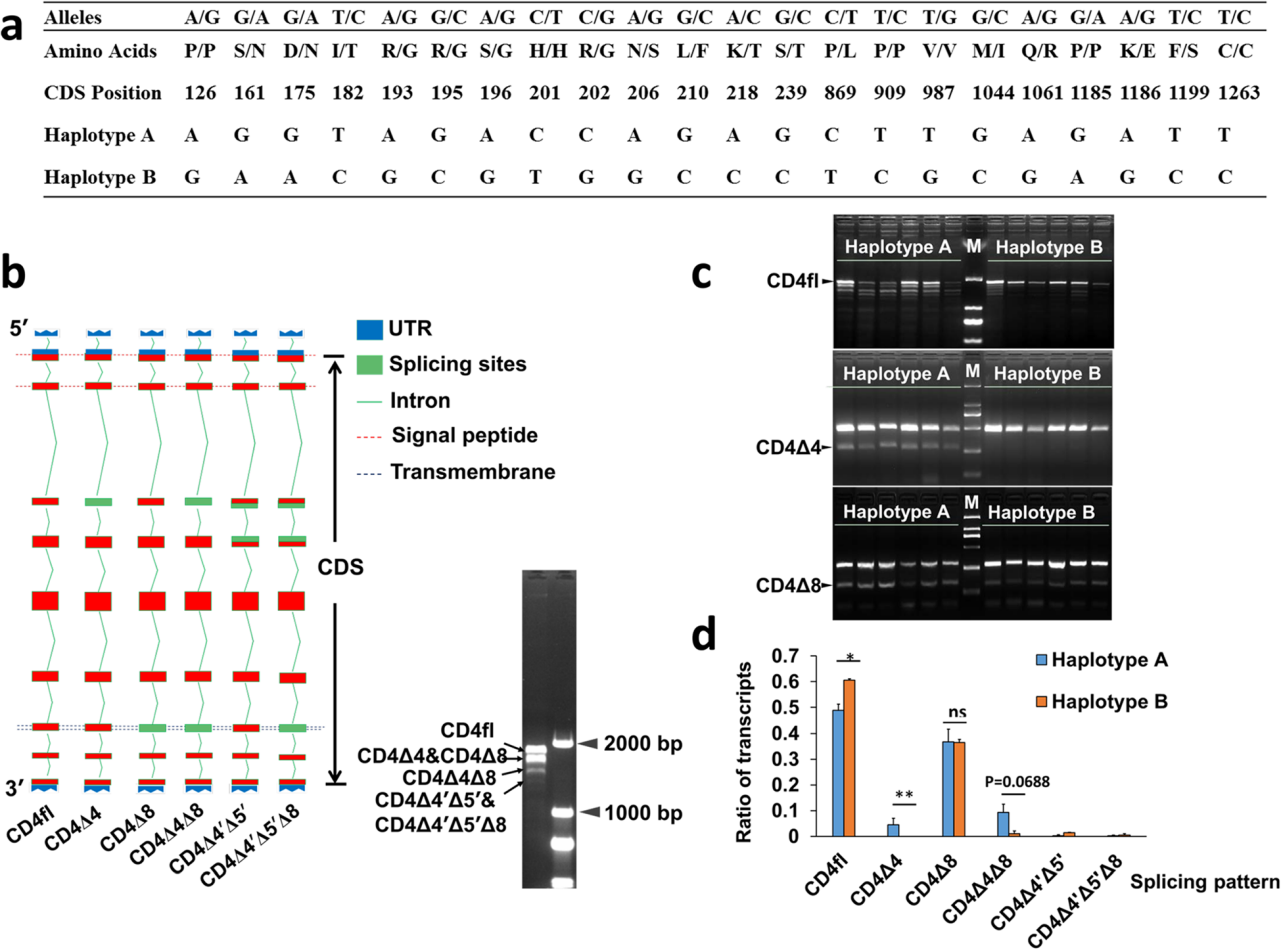
The identification of the mutation sites and transcripts of the CD4 gene in pigs. **a** The SNPs and two haplotypes of the CD4 gene. **b** The structure of different transcripts of the CD4 gene in pigs. **c** Detection of the abundance of different transcripts of porcine CD4 using RT-PCR. **d** Statistical analysis of the abundance of different transcripts between two haplotypes based on the number of different clones (haplotype A, *n* = 296; haplotype B, *n* = 218). *, *P* < 0.05; **, *P* < 0.01

To identify the haplotypes of the *CD4* gene in pigs, blood samples from 22 pigs were obtained for amplification and sequencing analysis of the *CD4* gene. In total, 22 SNPs in the CDS region were identified, which were linked completely and led to the formation of two haplotypes of the *CD4* gene (haplotypes A and B). Among them, 16 SNPs resulted in amino acid replacement (Fig. 1a). To investigate the functional differences of different haplotypes of the *CD4* gene, we examined the variable splicing of the *CD4* gene. The results showed that six transcripts could be formed by variable splicing, named CD4fl (full length), CD4Δ4 (exon 4 deletion), CD4Δ8 (exon 8 deletion), CD4Δ4Δ8 (exon 4 and 8 deletion), CD4Δ4′Δ5′ (partial deletion of both exons 4 and 5), and CD4Δ4′Δ5′Δ8 (partial deletion of exons 4 and 5, and total deletion of exon 8) (Fig. 1b). Furthermore, the abundance of the specific transcripts in haplotype A and B was analyzed. 514 single clones of the CDS fragment from three AB heterozygotes were selected randomly for sequencing. This result showed that CD4fl and CD4Δ8 were the transcripts in highest abundance. Moreover, the proportion of CD4fl in haplotype A was significantly lower than in haplotype B (0.49 ± 0.02 vs. 0.61 ± 0.01, *P* < 0.05), and the proportion of CD4Δ4Δ8 was higher in haplotype A than in B (*P* < 0.07). However, the CD4Δ4 transcript was only detected in haplotype A (*n* = 16/514) (Fig. 1c and d). These results indicated that the expression patterns between haplotypes A and B were different.

### The expression level and membrane localization were different between haplotypes a and B of the CD4 protein

**Fig. 2:**
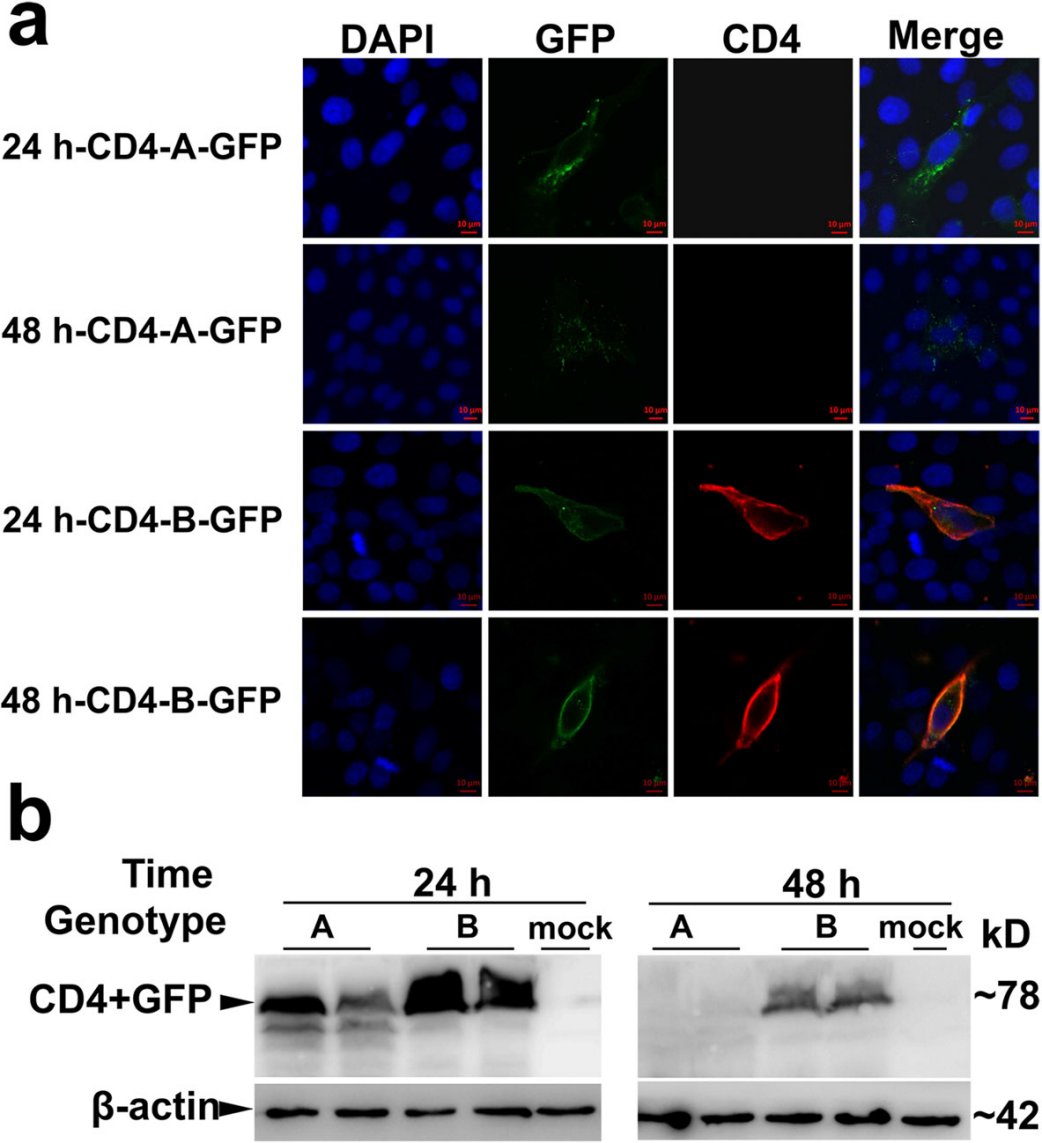
The expression pattern of the CD4 protein between two haplotypes in pigs. **a** Confocal images of the immune stain of GFP (green) and CD4 (red) proteins. Nucleus was stained with DAPI (blue). Scale bars: 10 μm. **b** Western blotting results of the CD4-GFP fusion protein at 24 h and 48 h post-transfection using GFP antibody. β-actin was used as internal control

To further explore the expression pattern of the *CD4* gene between haplotypes A and B, the translation level of the *CD4* gene was assessed in vitro. First, the CD4-GFP fusion protein vector was constructed. CD4-A-GFP and CD4-B-GFP fusion vectors were obtained by inserting the CDS regions of haplotypes A or B into pEGFP-n1 vectors, respectively. These two vectors were transfected into 3D4/21 cell lines. The fluorescence of GFP and the CD4 protein was detected by confocal microscopy at 24 h and 48 h post-transfection. The results showed that the GFP fluorescence signal in haplotype A was localized mainly in the cytoplasm, but the signal was observed primarily on the cell membrane in haplotype B. The fluorescence signal intensity of GFP in haplotype B was apparently stronger than that in haplotype A, especially at 48 h post-transfection. Moreover, immunofluorescence results showed that the fluorescence signal of the CD4 antibody was only detected in haplotype B (Fig. 2a).

Furthermore, the expression level of the CD4-GFP fusion protein was detected using a GFP antibody. The western blotting result showed that the expression level of the CD4-GFP fusion protein in haplotype B was obviously higher than that in haplotype A at both 24 h and 48 h post-transfection, and the protein levels of both haplotypes A and B at 24 h were higher than at 48 h post-transfection (Fig. 2b). Moreover, the CD4-A-flag and CD4-B-flag vectors were also constructed. The expression level of the CD4-Flag fusion protein was detected using a Flag antibody, and the results were consistent with those using CD4–GFP fusion vectors (Fig. S1). These results indicated that the protein level and membrane localization of the CD4 protein were different between haplotypes A and B.

### Pigs with genotypes AA and BB differed in their immune responses

**Fig. 3:**
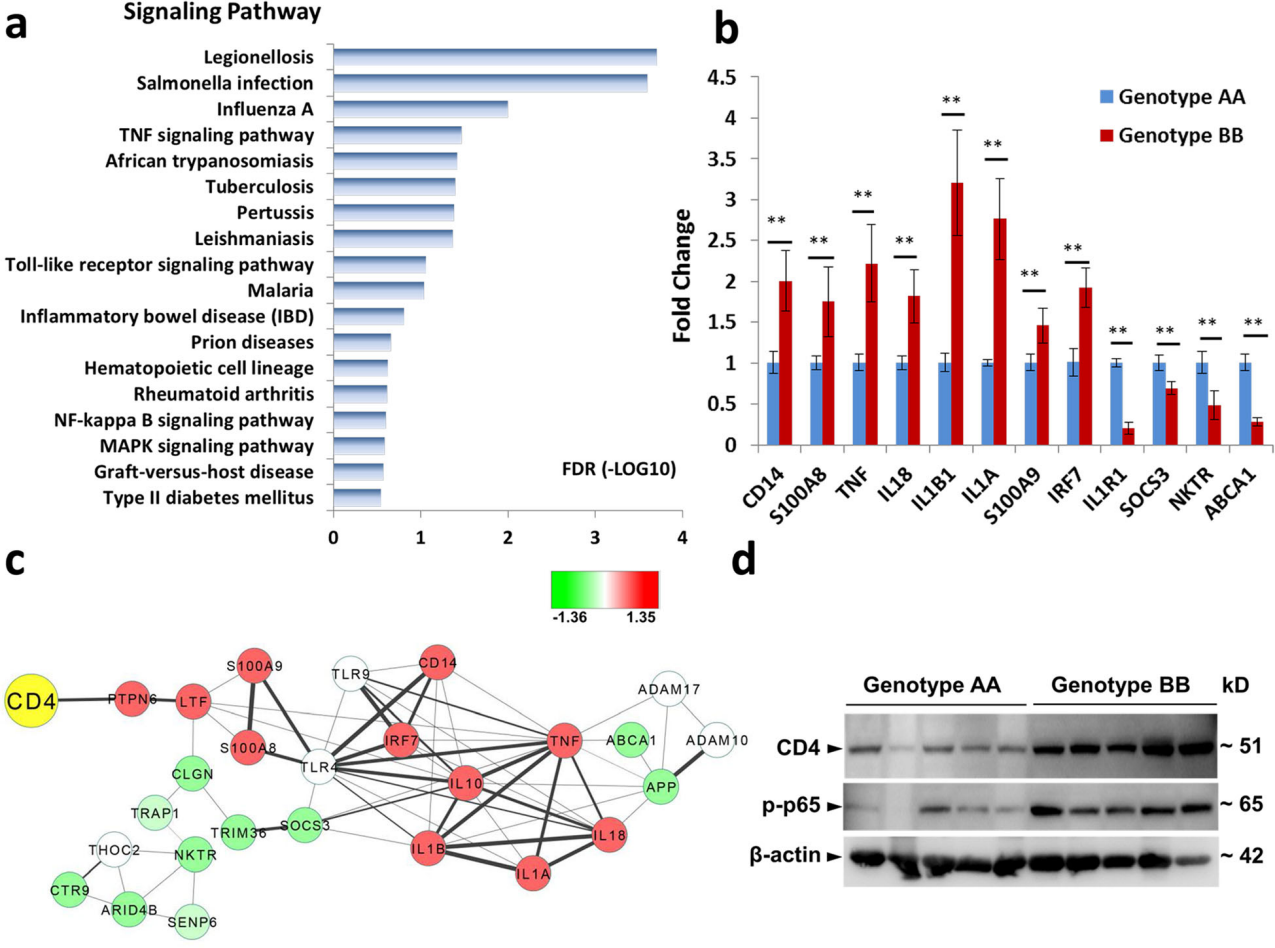
The functional verification of Haplotypes AA and BB at the individual level in pigs. **a** Functional analysis of differentially expressed genes between genotypes AA and BB in pigs (*n* = 10 for AA; *n* = 16 for BB). **b** Q-PCR validation of the differentially expressed genes (*n* = 6 for AA; n = 6 for BB). β-actin was used as the internal control for Q-PCR. Triplicate samples were analyzed for each treatment, and the results are presented as the mean ± s.e.m. **, *P* < 0.01. **c** The protein interaction network of DEGs. The red nodes represented genes that were up-regulated in individuals with genotype BB, and the green nodes represented genes that were down-regulated. The white nodes represented genes that were not significantly different between genotypes AA and BB. **d** Western blotting detection of the protein levels of CD4 and p-p65 of the two haplotypes (genotype AA, *n* = 5; genotype BB, n = 5) using anti-CD4 and anti-p65. β-actin was used as internal control

To study the functional differences between haplotypes A and B, the peripheral white blood cells from 10 AA and 16 BB pigs were selected for Affymetrix array analysis. According to the result, 180 differentially expressed genes (DEGs) were identified (FC ≥ 1.5, *P* < 0.05). Among them, 128 genes were up-regulated and 52 genes were down-regulated in pigs with genotype AA compared with genotype BB. The top 20 up- and down-regulated genes in genotype AA were listed in Table S1. Pathways were analyzed using the Kyoto Encyclopedia of Genes and Genomes database, and the result revealed that most significant enrichment pathways were related to immune responses, which included *Salmonella* infection, TNF, Toll-like receptor, NF-κB, etc. (Fig. 3a). Furthermore, 12 DEGs related to immune response were selected for further validation through Q-PCR (genotype AA: *n* = 6, genotype BB: n = 6) (Fig. 3b), which included eight up-regulated genes (*CD14*, *S100A8*, *Tnfα*, *Il18*, *Il1b1*, *Il1a*, *S100A9*, and *Irf7*) and four down-regulated genes (*Il1r1, Socs3, Nktr,* and *Abca1*). The Q-PCR results were positively correlated with the Affymetrix array results. The protein interaction network of DEGs was also analyzed using STRING and Cytoscape software. Almost all of the cytokines related to immune responses were up-regulated in genotype BB. However, cytokine signaling suppressors, such as SOCS3, were down-regulated in genotype BB (Fig. 3c). Moreover, western blotting analysis was performed using CD4 antibody and p65 antibody. The result showed that the protein level of phosphorylated p65, which was the activated subunit of NF-kappa B, was lower in genotype AA (*n* = 5) than in BB (n = 5) (Fig. 3d). These results indicated that the function of the *CD4* gene in immune responses was different between genotypes AA and BB.

### Three functional mutations determined the functional differences between haplotypes a and B

**Fig. 4:**
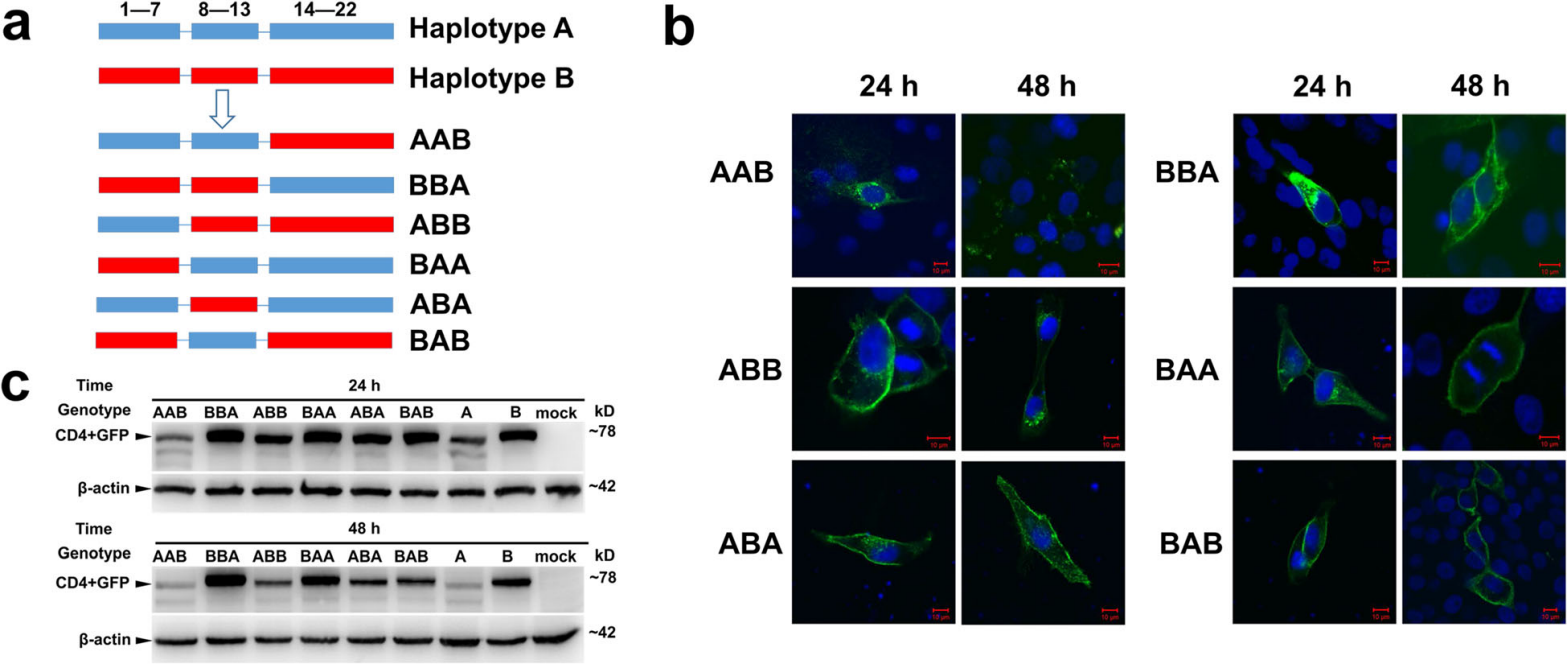
The identification of the key regions in the CD4 gene that led to the functional difference between haplotypes A and B in pigs. **a** The different chimeras of haplotypes A and B of the CD4 gene. Haplotypes A (blue) and B (red) were divided into three clusters that covered 1st–7th, 8th–13th, and 14th–22nd SNPs. AAB and BBA represented for the mutation of 14th–22nd SNPs of haplotypes A and B. ABB and BAA represented for the mutation of 1st–7th SNPs. ABA and BAB represented for the mutation of 8th–13th SNPs. **b** Confocal images of the immune stain of the GFP (green) protein. Nucleus was stained with DAPI (blue). Scale bars: 10 μm. **c** Western blotting results of the CD4-GFP fusion protein at 24 h and 48 h post-transfection using GFP antibody. β-actin was used as internal control

To explore the specific SNP sites that affected functional differences between haplotypes A and B, the functional mutations in the *CD4* gene were identified. First, the 22 SNPs were divided into three clusters, called cluster 1 (1st–7th SNPs), cluster 2 (8th–13th SNPs), and cluster 3 (14th–22nd SNPs). Six chimeras of haplotypes A and B were constructed, named as AAB, BBA, ABA, BAB, ABB, and BAA (Fig. 4a). These six chimeras were inserted into the pEGFP-n1 vector and then transfected into 3D4/21 cell lines. Subsequently, the fluorescence signal and expression level of the GFP protein were detected at 24 h and 48 h post-transfection. Immunofluorescence results showed that the AAB chimera had an expression pattern similar to CD4-A-GFP, whereas the other chimeras were similar to CD4-B-GFP (Fig. 4b). Western blotting results also showed that the expression level of AAB was comparable with CD4-A-GFP, but was lower than that of the other five chimeras and CD4-B-GFP (Fig. 4c). Based on these results, we speculated that the key functional mutations were located in the regions of cluster 1 and cluster 2.

**Fig. 5:**
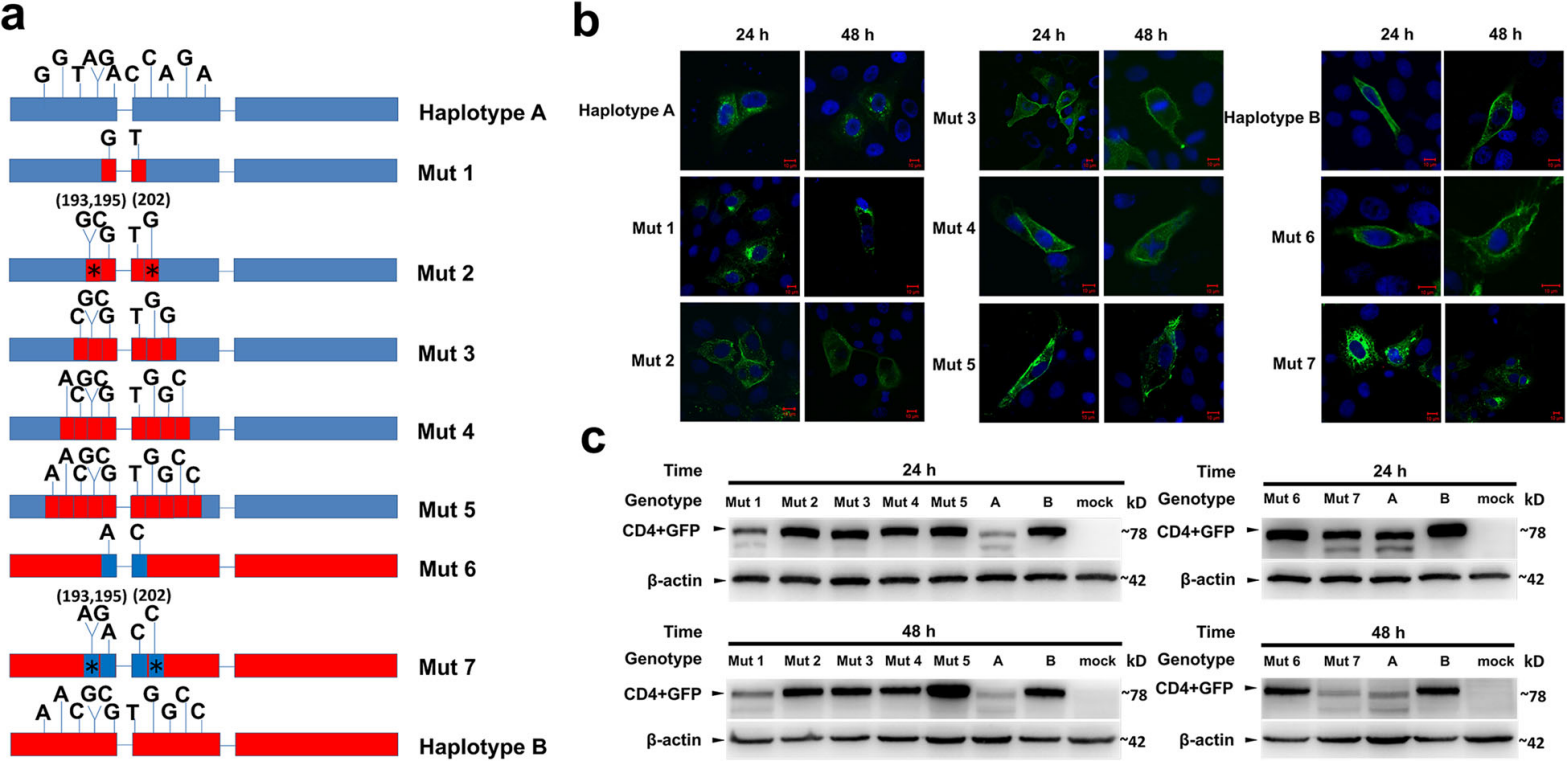
The identification of the key mutation sites that led to the functional difference between haplotypes A and B in pigs. **a** Model of mutant vectors of the CD4 gene. Mut 1, Mut 2, Mut 3, Mut 4, and Mut 5 indicated that 2, 5, 7, 9, and 11 SNPs mutated from haplotype A to haplotype B, respectively. Mut 6 and Mut 7 indicated that 2 and 5 SNPs mutated from haplotype B to haplotype A, respectively. Red and blue squares indicate the amino acids that corresponded with the SNPs. All mutants were inserted into a pEGFP-n1 vector for the construction of the GFP fusion protein. **b** Confocal images of the immune stain of GFP (green) protein. Nucleus was stained with DAPI (blue). Scale bars: 10 μm. **c** Western blotting results of the CD4-GFP fusion protein at 24 h and 48 h post-transfection using GFP antibody. β-actin was used as internal control

Furthermore, the specific SNPs in cluster 1 and cluster 2 were studied by constructing a series of mutation vectors. First, the two adjacent SNPs (7th and 8th) were mutated (from AC in A to GT in B, involved two amino acids, and marked in red in Fig. 5a), and the mutation was named “Mut 1”. Second, five SNPs (from the 5th to the 9th) were mutated from AGACC in haplotype A to GCGTG in haplotype B (involved four amino acids, and marked in red in Fig. 5a), and the mutation was named “Mut 2”. Furthermore, seven (from the 4th to the 10th, involved six amino acids, and marked in red in Fig. 5a), nine (from the 3rd to the 11th, eight amino acids, and marked in red in Fig. 5a), and 11 (from the 2nd to the 12th, ten amino acids, and marked in red in Fig. 5a) SNPs were mutated from haplotype A to B, and the mutations were named “Mut 3, Mut 4, and Mut 5”, respectively (Fig. 5a). These mutant vectors were transfected into 3D4/21 cell lines. Then, the fluorescence signal and expression level of the GFP protein were detected at 24 h and 48 h post-transfection. Immunofluorescence results showed that CD4 proteins were localized in the cytoplasm in cells transfected with Mut1 or haplotype A at 24 h and 48 h post-transfection. The expression pattern and localization of the CD4 protein in cells transfected with other mutational constructs were similar to that of haplotype B both at 24 h and 48 h (Fig. 5b). Western blot results indicated that the CD4 protein level in cells transfected with Mut1 was similar to haplotype A, but lower than that of haplotype B and other mutational constructs (Fig. 5c).

Moreover, the specific bases of haplotype B were also mutated to that of haplotype A, and the mutations were named “Mut 6” (GT to AC at the 7th and 8th sites that involved two amino acids, and mutation sites were marked in blue) and “Mut 7” (GCGTG to ACACC from the 5th to the 9th sites that involved two amino acids, and the mutation sites were marked in blue)”. Immunofluorescence results showed that the CD4 protein in cells transfected with Mut 6 were localized on cell membranes, which was similar to haplotype B, but the CD4 protein in cells transfected with Mut 7 mainly localized in the cytoplasm, which was similar to haplotype A (Fig. 5b). Western blotting results showed that the protein expression level of the CD4 protein in cells transfected with Mut 6 was comparable with haplotype B, but it was much higher than that of haplotype A and Mut 7, especially at 48 h post-transfection (Fig. 5c). These results indicated that the 193A/G SNPs, 195G/C SNPs, and 202C/G SNPs in the CDS region could influence the expression level and membrane localization of the CD4 protein.

**Fig. 6:**
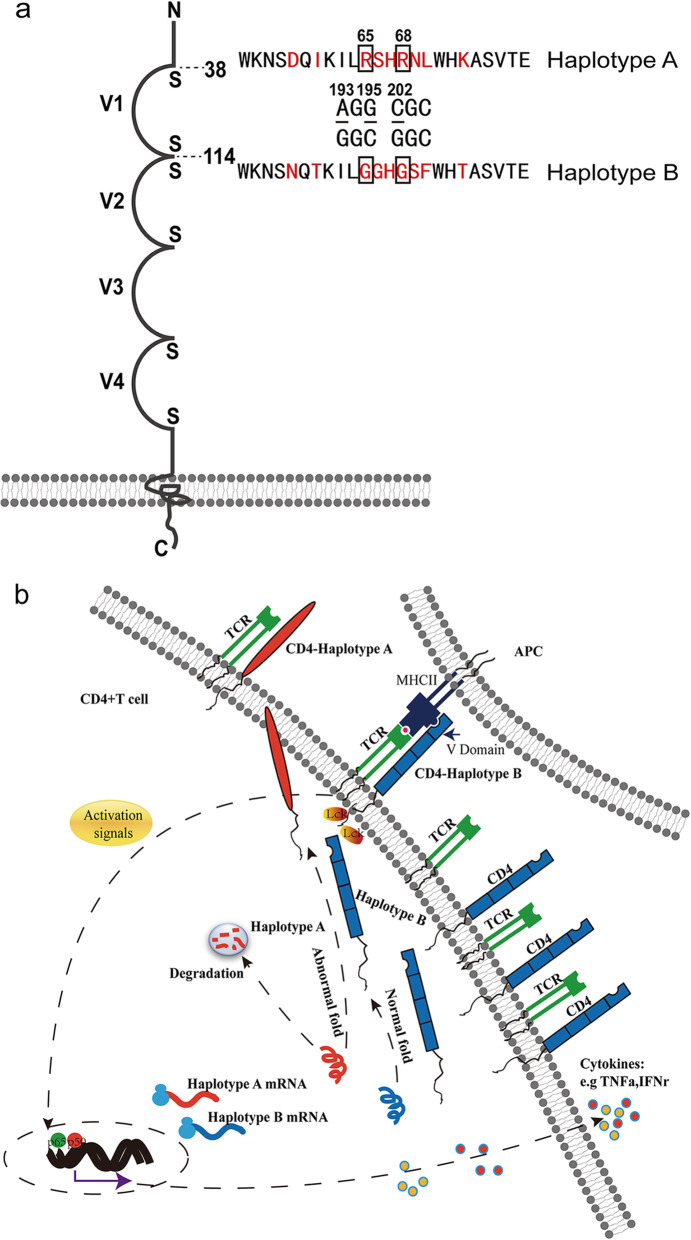
The localization of two key amino acids and the schema graph of the function of CD4 protein in the immune response of T cells. **a** The structure of the CD4 protein domain in pigs, which included four extracellular domains, one trans-membrane domain, and one intercellular domain. The three key SNPs (193A/G, 195G/C, and 202C/G) led to two amino acid conversions (65R/G and 68R/G), and these two amino acids were in the IgV1 domain. **b** The schema graph of the function of the CD4 protein in the immune response

In addition, 193A/G SNPs, 195G/C SNPs, and 202C/G SNPs were further considered. These three SNPs were mutated one by one, and the results showed that all of them affected the expression level and localization of the CD4 protein (Fig. S2). In addition, we found that these three SNPs led to the replacement of 65th and 68th amino acids in the CD4 protein, and these two amino acids were localized at the IgV1 domain (Fig. 6a). Therefore, these three SNPs (193A/G, 195G/C, and 202C/G, which involved 65R/G and 68R/G amino acids replacement) were important for the expression pattern and function in the immune response of the CD4 protein.

### The distribution of a and B alleles was different among pig breeds

This study further investigated the distribution of A and B alleles in different pig breeds. Bases on the mutation results, the 193A/G SNP, 195G/C SNP, and 202C/G SNP affected the expression and function between different haplotypes of the *CD4* gene. Therefore, the distribution of these three linked SNPs was researched in four group of pig breeds, which included ten Chinese indigenous breeds (*n* = 334), four western commercial breeds (*n* = 312), one European wild boar population (*n* = 47), and one Asian wild boar population (*n* = 40). The result showed that the frequency of allele A in Chinese breeds (q = 0.445) was higher than in western commercial breeds (q = 0.008), the European wild boar population (q = 0), or the Asian wild boar population (q = 0.088) (Table 1), while the distribution of allele B was just the opposite.

**Table 1:** Allele distribution of the three linked functional mutations in different breeds of pigs

Population	Breed	Number	Genotype			Allele A frequency	Allele B frequency
			AA	AB	BB		
Chinese breeds	Erhualian	17	10	7	0	0.445	0.555
	Luchuan	55	34	21	0		
	Longlin	39	8	13	18		
	Dongshan	22	0	8	14		
	Xiaomeishan	11	5	6	0		
	Wuzhishan	14	6	6	2		
	Dahuabai	17	8	7	2		
	Lantang	12	2	9	1		
	Enshi Black	69	8	40	21		
	Tongcheng	78	0	18	60		
Western breeds	Duroc	34	0	0	34	0.008	0.992
	Large White	254	0	5	249		
	Landrace	16	0	0	16		
	Pietrain	8	0	0	8		
European wild boar		47	0	0	47	0	1
Asian wild boar		40	1	5	34	0.088	0.912

## Discussion

The CD4 protein was confirmed to play an important role in the development of T cells and the immune response [19], and the *CD4* gene was an important candidate for immune-related diseases in humans and pigs [13, 20, 21]. The *CD4* gene interacted with the *MHC* gene, and both of them were rich in SNP sites [22]. Previous studies mentioned that immune-related genes were rich in SNPs, and they could be beneficial for adaptive immune responses by the host [23–25].

In this study, 22 SNPs were found in the CDS region of the *CD4* gene, which led to the conversion of amino acids and the formation of two haplotypes. Matsubara et al. also divided the *CD4* gene into two haplotypes in miniature pigs by SNP analysis, but it is different from the SNPs screened out in this study [18]. Furthermore, we investigated the functional differences between the two haplotypes. Interestingly, there was no significant difference at the transcription level between haplotypes A and B (Fig. S3), but the translation level was significant difference in vitro and in vivo. Therefore, we postulated that the post-transcription regulation mainly caused the protein level difference between the two haplotypes.

The CD4 protein participated in the representation of antigen through interaction with the MHCII molecule and played an important role in the immune response [19]. In this study, we found that the expression level of the CD4 protein was significantly different between haplotypes A and B. We suspected that this led to differences in the immune response in pigs. As expected, transcriptome analysis indicated that most of the DEGs were enriched in inflammatory and immune-related pathways, which included the TLR, TNF, and NF-κB classic immune regulatory signaling pathways. Moreover, most cytokines related to immune responses were down-regulated in genotype AA. Furthermore, TNFα, IL1B1, and TLR4 were the center nodes in the analysis of protein interaction network, and the expression levels of *Tnfα* and *Il1b1* genes were significantly lower in haplotype A than in haplotype B. Moreover, western blotting results showed that the expression level of phosphorylated p65 was down-regulated in genotype AA. It is known that phosphorylated p65 could positively regulate TNFα and IL1B1 at the transcriptional level [26, 27]. These results indicated that there were significant differences between the two haplotypes of the *CD4* gene in the immune response.

This study demonstrated that the formation of two haplotypes of the *CD4* gene was due to the existence of 22 fully linked SNPs in the CDS region, among which three key sites determined the functional conversion between haplotypes A and B. In addition, these three linked SNPs led to the mutation of two amino acids in the IgV1 domain of the CD4 protein (Fig. 6a), which is a domain that was very important for the function of the CD4 protein in the immune response [28]. IgV1 was the key domain in the binding of CD4 protein to MHCII [29, 30] and to gp120 [7, 31, 32]. Therefore, we hypothesized that the IgV1 domain of the CD4 protein in haplotype A was dysfunctional, which led to the enhanced degradation of the CD4 protein. However, the CD4 protein of haplotype B had a fully functional structural domain, which transmitted the activation signal from antigen presenting cells (APC) to T cells that stimulated the release of immune response factors by activating the expression of p65 (Fig. 6b).

In addition, we found that the distribution of alleles A and B was different among different pig breeds. The *CD4* gene appeared as allele B in almost all western commercial pigs and wild pigs, and as allele A in about 50% of Chinese domestic pigs. We believe that Chinese domestic pigs experienced a selection process that resulted in an increase in the frequency of allele A. The *CD4* gene may be one of the candidates for this difference. Previous studies confirmed that Chinese indigenous breeds had different immune responses compared with western commercial pigs due to a selection sweep [33, 34]. Interestingly, we found a small number of AB heterozygotes in Large White pigs (Table 1). During breeding, Chinese pigs were used for Large White breed cultivation [34]. Thus, we suspected that allele A in the Large White pig breed originated from Chinese pig breeds.

## Conclusion

Combining these results, we concluded that the difference in the immune response between haplotypes A and B was due to the presence of three SNPs (193A/G, 195G/C, and 202C/G) in the CDS region of the *CD4* gene. Further, these three SNPs may be used for animal breeding or as therapeutic targets of immune-related diseases.

## Methods

### Cells and samples

The white blood cells were obtained from the F2 population of Duroc × Erhualian. The total RNA, DNA, and protein used in this study were extracted from the white blood cells. 3D4/21 cells were bought from ATCC (https://www.atcc.org/). DNA used for allele frequency analysis was stored in our lab.

### Transcriptome and functional analysis of the differentially expressed genes

Gene expression patterns of white blood cells were detected by using Affymetrix GeneChip. Haplotype AA (*n* = 10) and BB individuals (*n* = 16) were selected for analysis of differentially expressed genes. The details of the analysis were shown in our previous studies [35, 36]. Briefly, all raw probe-level microarray data were normalized by the Robust Multichip Average method with the bioconductor AFFY package (www.bioconductor.org). The differentially expressed genes (*P* < 0.05, FC ≥ 1.5) were identified using the LIMMA tool. Two-way hierarchical clustering analysis was performed after identifying the differentially expressed genes. The signaling pathways of differentially expressed genes were analyzed using DAVID tools (https://david.ncifcrf.gov/). The interaction of the differentially expressed genes was analyzed using STRING (https://string-db.org/) and the network figure was drawn using Cytoscape 3.5.1 (http://www.cytoscape.org/).

### Identification of the transcripts

The RNA of three individuals with genotype AB of the *CD4* gene was extracted. RT-PCR was performed to amplify the exon regions of the *CD4* gene. The reverse transcription PCR (RT-PCR) products contained the full length coding regions and partial 3′- and 5′-UTR regions of the *CD4* gene. The products were cloned in the pMD-19 T vector and transfected into *E. coli* DH5α. A total of 514 clones were sequenced. The different transcripts of the *CD4* gene and their frequencies were calculated. A T-test was used for significance analysis. The primers used for RT-PCR are listed in Table S2.

### Quantitative PCR (Q-PCR)

The RNA in peripheral white blood cells of F2 pigs with genotype AA (*n* = 6) and BB (n = 6) were selected randomly for Q-PCR analysis. The quality of total RNA was assessed using Nano Drop2000 (ThermoFisher Scientific, Waltham, MA, USA). In brief, 1 μg of the RNA of each sample was used to synthesize cDNA using a PrimeScript™ RT reagent Kit with gDNA Eraser (Takara, Tokyo, Japan). THUNDERBIRD SYBR qPCR Mix (TOYOBO, Japan) was used for Q-PCR, and the results were monitored using a CFX384 Real-Time PCR Detection System (Bio-Rad, USA). The primers are listed in Table S3. T-test was used for statistical analysis.

### Construction of expression vectors, cell culture, and transfection

pEGFP-n1 (Clontech, USA) and pCDNA3.1^+^ (Invitrogen, USA) vectors were used to construct CD4-GFP fusion protein and CD4-flag expression vectors (Table S4 and Table S5). The 3D4/21 cell line was stored previously in 7% dimethyl sulfoxide (DMSO) in our lab. The cells were gently thawed in a 37 °C water bath, and then DMSO was removed and discarded. The cells were cultured in RPMI 1640 (Gibco, USA) supplemented with 10% FBS and 1% MEM NEAA (Gibco, USA) and cultured routinely at 37 °C with 5% CO_2_. For sub-cultivation, cells were rinsed with 0.25% trypsin–0.53 mM EDTA solution. All the constructions were transfected into 3D4/21 cells using Lipofectamine 2000 (Invitrogen, USA) according to the manufacturer’s recommendations.

### Immunofluorescence

Cells were seeded on chamber slides. The cells were then fixed for 10 min in 4% paraformaldehyde. After fixing, the cells were blocked for 2 h in 3% BSA and 10% FBS. The cells were incubated at 4 °C overnight with 1:100 Phycoerythrin -conjugated Mouse anti-pig CD4a (BD, #559586, USA) or 1:2000 anti-flag antibody (ABclonal, #AE005, China). After washing with PBS, the cells were stained with nuclear-specific DAPI stain. After staining, the cells were photographed using a laser scanning confocal microscope (ZEISS, Germany, LSM 510 META).

### Western blotting

3D4/21 cells were plated in six-well culture dishes and transfected with target vectors when they converged to approximately 70%. After 24 h and 48 h, the total proteins in the cells were extracted using RIPA buffer (Thermo Fisher Scientific, New York, USA, #89900). The proteins were diluted in SDS loading buffer and then heated for 10 min at 100 °C for denaturation. The proteins were electrophoresed through 12% SDS-PAGE and then transferred to a nitrocellulose filter. Membranes were blocked in 5% non-fat milk for 1 h at room temperature and incubated with 1:1000 anti-GFP antibody (ABclonal, AE012), 1:2000 anti-flag antibody (ABclonal, AE005), 1:1000 anti-CD4 antibody (Abcam, UK, 25804), 1:1000 anti-β-actin antibody (Beyotime, Shanghai, China, AF0003), or 1:1000 anti-p-p65 antibody (CST, USA, 4025) at 4 °C overnight. Membranes were incubated with 1:1000 horseradish peroxidase-labeled anti-rabbit-IgG secondary antibody (Beyotime, Shanghai, China, A0208) or anti-mouse-IgG secondary antibody (Beyotime, Shanghai, China, A0216) for 1 h at room temperature. Immunodetection was performed by an Image Quant LAS4000 mini (GE, USA). The gray values of the protein bands were measured using ImageQuantTL software (GE, USA), and β-actin protein levels were used as internal control.

## Supplementary Information

**Additional file 1: Figure S1**. The expression pattern of the CD4 protein in two haplotypes using a PCDNA3.1 + −CD4–flag fusion vector.

**Additional file 2: Figure S2**. The identification of the key mutation sites that led to the functional difference between haplotypes A and B.

**Additional file 3.** Q-PCR results of the expression change of the CD4 gene in genotypes AA and BB.

**Additional file 4: Table S1**. Top 20 of up-regulated genes and down-regulated genes in pigs with genotype AA.

**Additional file 5: Table S2**. Primers used for haplotype identification in this study.

**Additional file 6: Table S3**. Primers used for Q-PCR in this study.

**Additional file 7: Table S4** Primers used for vector construction in this study.

**Additional file 8: Table S5** Primers used for vector construction in this study.

## Data Availability

The datasets used and analyzed during the current study available from the corresponding author on reasonable request.
