# Supplementary figures and images for: Three functional mutation sites affect the immune response of pigs through altering the expression pattern and IgV domain of the CD4 protein
Source: BMC Mol Cell Biol. 2020 Dec 9;21:91. doi: 10.1186/s12860-020-00333-7 (PMC7724863; doi:10.1186/s12860-020-00333-7)

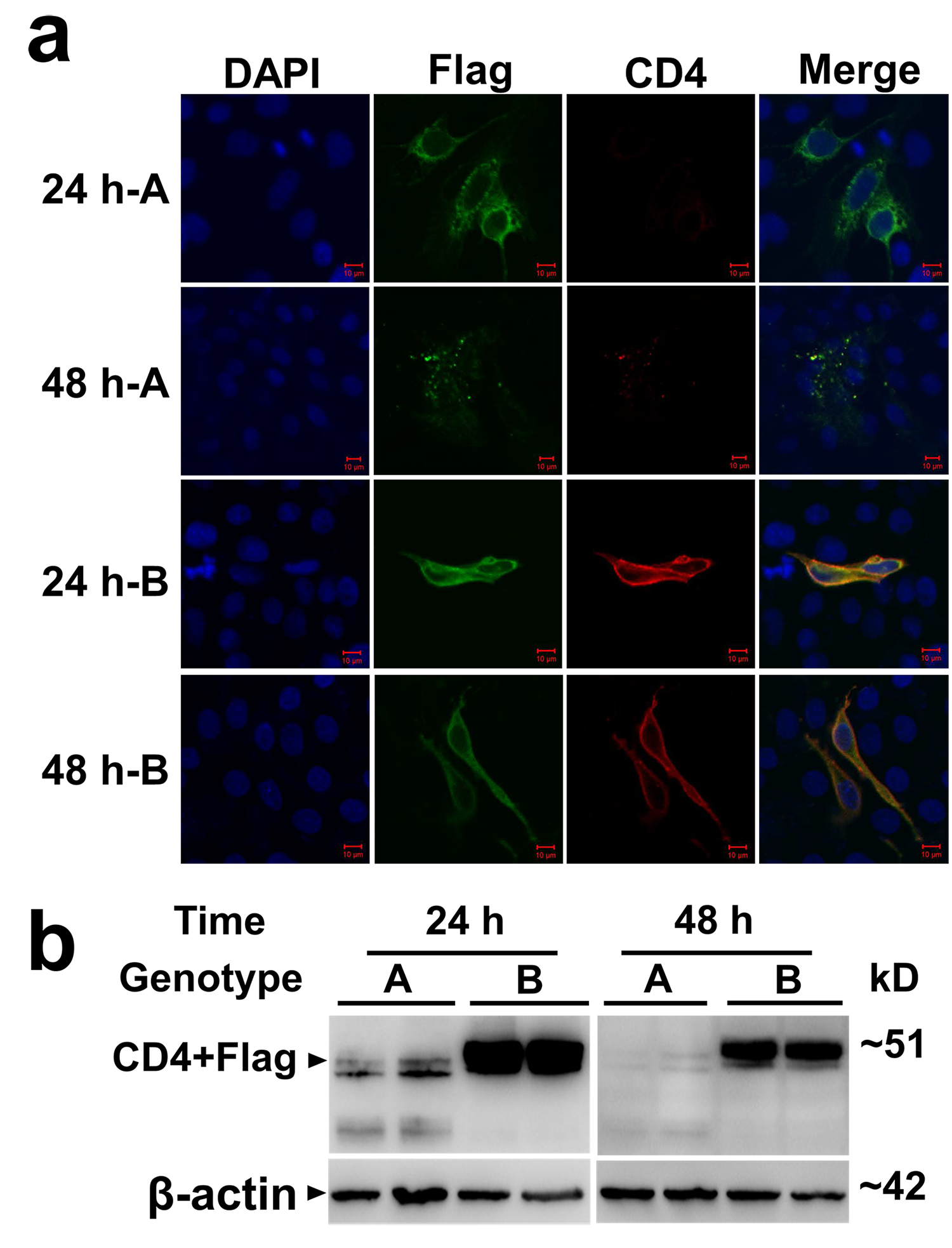

Supplement: Supplementary file 1 — Additional file 1: Figure S1. The expression pattern of the CD4 protein in two haplotypes using a PCDNA3.1 + −CD4–flag fusion vector. [file 12860_2020_333_MOESM1_ESM.tif]

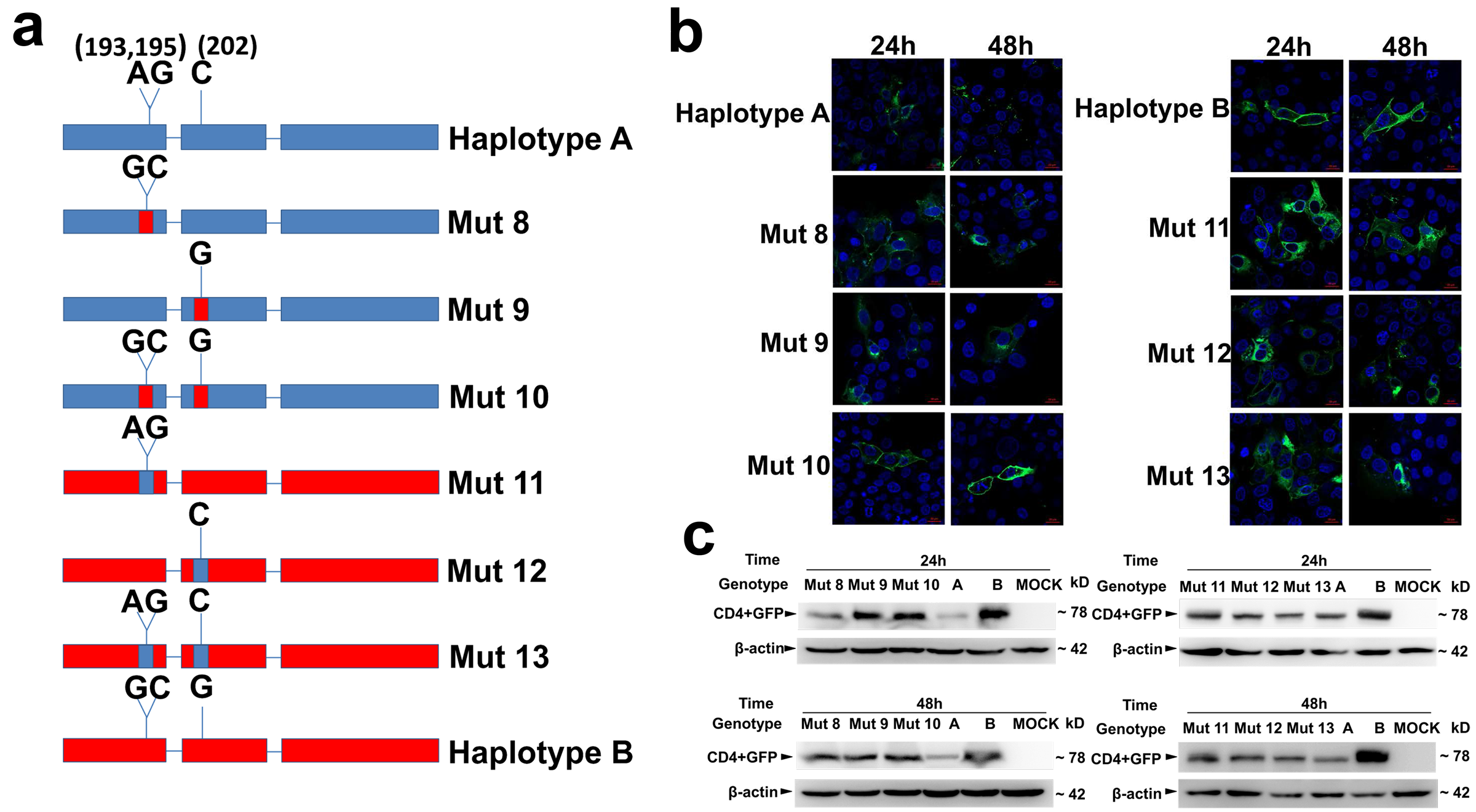

Supplement: Supplementary file 2 — Additional file 2: Figure S2. The identification of the key mutation sites that led to the functional difference between haplotypes A and B. [file 12860_2020_333_MOESM2_ESM.tif]

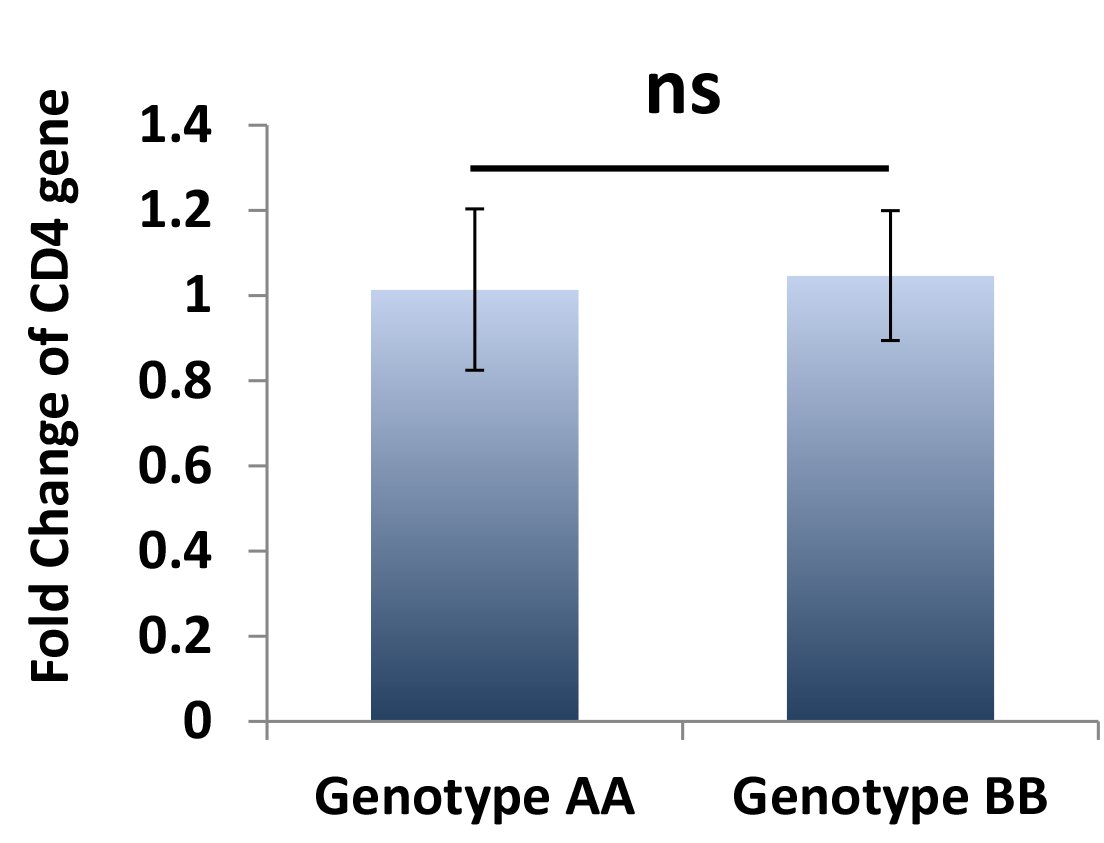

Supplement: Supplementary file 3 — Additional file 3. Q-PCR results of the expression change of the CD4 gene in genotypes AA and BB. [file 12860_2020_333_MOESM3_ESM.tif]
