# Supplementary material for: Three functional mutation sites affect the immune response of pigs through altering the expression pattern and IgV domain of the CD4 protein
Source: BMC Mol Cell Biol. 2020 Dec 9;21:91. doi: 10.1186/s12860-020-00333-7 (PMC7724863; doi:10.1186/s12860-020-00333-7)
Supplement: Supplementary file 4 — Additional file 4: Table S1. Top 20 of up-regulated genes and down-regulated genes in pigs with genotype AA. [file 12860_2020_333_MOESM4_ESM.docx]

Table S1. Top 20 of up-regulated genes and down-regulated genes in pigs with genotype AA

| Up-regulated Genes | LogFC | P-Value | Down-regulated Genes | LogFC | P-Value |
| --- | --- | --- | --- | --- | --- |
| DDX21 | 1.35 | <0.01 | EGR1 | -1.36 | 0.01 |
| SMC6 | 1.13 | <0.01 | CLYBL | -1.34 | 0.03 |
| SLC12A8 | 1.11 | 0.01 | RHOB | -1.25 | 0.01 |
| ABCA1 | 1.11 | <0.01 | CXCL2 | -1.25 | 0.02 |
| RSF1 | 1 | 0.01 | ACTB | -1.2 | 0.01 |
| TCF4 | 1 | <0.01 | LTF | -1.16 | <0.01 |
| USP16 | 0.98 | <0.01 | CD14 | -1.13 | 0.02 |
| CTCF | 0.95 | <0.01 | S100A8 | -1.06 | 0.02 |
| NP_055907 | 0.93 | 0.03 | TNFΑ | -0.98 | <0.01 |
| SMC3 | 0.93 | <0.01 | CAMP | -0.97 | 0.03 |
| ARID4B | 0.92 | 0.03 | IL18 | -0.96 | 0.03 |
| LRRIQ1 | 0.91 | <0.01 | IL1B1 | -0.96 | 0.04 |
| DHX29 | 0.91 | 0.01 | IRF7 | -0.95 | 0.02 |
| CALD1 | 0.9 | 0.04 | IER3 | -0.93 | <0.01 |
| NKTR | 0.89 | 0.02 | EDN1 | -0.93 | 0.03 |
| HPGD | 0.88 | <0.01 | FMNL2 | -0.92 | 0.03 |
| ILF3 | 0.88 | <0.01 | MXD1 | -0.91 | <0.01 |
| SLC7A11 | 0.88 | 0.02 | MS4A8B | -0.9 | 0.04 |
| GLS | 0.88 | 0.04 | CCL4L | -0.89 | 0.02 |
| CLGN | 0.87 | <0.01 | IER5 | -0.84 | 0.01 |
