# Supplementary material for: Three functional mutation sites affect the immune response of pigs through altering the expression pattern and IgV domain of the CD4 protein
Source: BMC Mol Cell Biol. 2020 Dec 9;21:91. doi: 10.1186/s12860-020-00333-7 (PMC7724863; doi:10.1186/s12860-020-00333-7)
Supplement: Supplementary file 5 — Additional file 5: Table S2. Primers used for haplotype identification in this study. [file 12860_2020_333_MOESM5_ESM.docx]

Table S2. Primers used for haplotype identification in this study

| Name | Sequence（5'-3'） | TM（°C） | Length（bp） |
| --- | --- | --- | --- |
| CD4-Hyp-S | CTCGGCAAAACCACAATG | 60 | 1593 |
| CD4-Hyp-A | GGAAAAGGGAAAGAGGAAGAAAG |  |  |
