# Supplementary material for: Three functional mutation sites affect the immune response of pigs through altering the expression pattern and IgV domain of the CD4 protein
Source: BMC Mol Cell Biol. 2020 Dec 9;21:91. doi: 10.1186/s12860-020-00333-7 (PMC7724863; doi:10.1186/s12860-020-00333-7)
Supplement: Supplementary file 6 — Additional file 6: Table S3. Primers used for Q-PCR in this study. [file 12860_2020_333_MOESM6_ESM.docx]

Table S3. Primers used for Q-PCR in this study

| Name | Sequence（5'-3'） | TM（°C） | Length（bp） |
| --- | --- | --- | --- |
| CD4-S | CAAAAGTCTCTCCAGAACCTCAAG | 60 | 193 |
| CD4-A | CCTCACAGGTCACTTCGTTCTTAG |  |  |
| CD14-S | CAGACTCCGTAATGTGTCGTGG | 60 | 173 |
| CD14-A | GATTGTCAGATAGGTCCAGGGT |  |  |
| S100A8-S | GGATCTGGAGAGTGCCATTAAC | 60 | 171 |
| S100A8-A | ATGTCCAGCTCTTTGAACCAGG |  |  |
| TNFα-S | GGACTCAGATCATCGTCTCAAAC | 60 | 192 |
| TNFα-A | GAAGAGGACCTGGGAGTAGATGA |  |  |
| IL18-S | GTTTGAGGATATGCCTGATTCTG | 60 | 122 |
| IL18-A | TCTTACACTGCACAGAGATGGTT |  |  |
| IL1B1-S | CTACCCTCTCCAGCCAGTCTTC | 60 | 156 |
| IL1B1-A | TGGGTGCAGCACTTCATCTCT |  |  |
| IL1A-S | TCCAGAGCAACATGAAATACAACTT | 60 | 196 |
| IL1A-A | GTCACAGGAAGTTGCGAATCATC |  |  |
| S100A9-S | GCAGCATAGAAACCATTATCAACAT | 60 | 122 |
| S100A9-A | CTTGAGAAAGTTTGGCAGCTCTT |  |  |
| SOCS3-S | CGAGAAGATCCCTCTGGTGTTG | 60 | 151 |
| SOCS3-A | GTACTGGTCCAGGAACTCCCG |  |  |
| NKTR-S | TATAAAGGTTCTACATTCCATCGTG | 60 | 144 |
| NKTR-A | CGCTCTGTCATGTTTGAGAATAA |  |  |
