# Supplementary material for: Three functional mutation sites affect the immune response of pigs through altering the expression pattern and IgV domain of the CD4 protein
Source: BMC Mol Cell Biol. 2020 Dec 9;21:91. doi: 10.1186/s12860-020-00333-7 (PMC7724863; doi:10.1186/s12860-020-00333-7)
Supplement: Supplementary file 7 — Additional file 7: Table S4 Primers used for vector construction in this study. [file 12860_2020_333_MOESM7_ESM.docx]

Table S4 Primers used for vector construction in this study

| Name | Sequence（5'-3'） | TM（°C） | Length（bp） |
| --- | --- | --- | --- |
| pEGFP-CD4-S | CCGCTCGAGCAATGGACCCAGGAA | 60 | 1392 |
| pEGFP-CD4-A | AAAACTGCAGGGTGAGGGAATAGT |  |  |
| pCDNA-CD4-Flag-F | CGGGGTACCATGGACCCAGGAACCTCTCT | 60 | 1416 |
| pCDNA-CD4-Flag-R | CCGCTCGAGTTACTTATCGTCGTCATCCTTGTAATCGGTGAGGGAATAGTTCTTCTGTT |  |  |
