# Supplementary material for: Three functional mutation sites affect the immune response of pigs through altering the expression pattern and IgV domain of the CD4 protein
Source: BMC Mol Cell Biol. 2020 Dec 9;21:91. doi: 10.1186/s12860-020-00333-7 (PMC7724863; doi:10.1186/s12860-020-00333-7)
Supplement: Supplementary file 8 — Additional file 8: Table S5 Primers used for vector construction in this study. [file 12860_2020_333_MOESM8_ESM.docx]

Table S5 Primers used for vector construction in this study

| Name | Sequence（5'-3'） | TM（°C） | Length（bp） |
| --- | --- | --- | --- |
| Forward-AAB&BBA-F | CCGCTCGAGCAATGGACCCAGGAACCTCTCT | 60 | 609 |
| Forward- AAB&BBA-R | TACTCCTGAACACCAGTGTCTTCTGGTCCTGGGAGACAGT |  |  |
| Behind- AAB&BBA-F | GACACTGGTGTTCAGGAGTA | 60 | 803 |
| Behind- AAB&BBA-R | AAAACTGCAGGGTGAGGGAATAGTTCTTCTGTTG |  |  |
| Forward-ABB&BAA-F1 | CCGCTCGAGCAATGGACCCAGGAACCTCTCT | 60 | 257 |
| Forward- ABB&BAA-R1 | GCGAGAGGTCAACTCGGTAACTGAGGCTGTGTGCCAGAAGCTGCCATGAC |  |  |
| Behind- ABB&BAA-F1 | TTACCGAGTTGACCTCTCGC | 60 | 1155 |
| Behind- ABB&BAA-R1 | AAAACTGCAGGGTGAGGGAATAGTTCTTCTGTTG |  |  |
| Forward-ABB&BAA-F2 | CCGCTCGAGCAATGGACCCAGGAACCTCTCT | 60 | 257 |
| Forward- ABB&BAA-R2 | GCGAGAGCTCAACTCGGTAACTGAGGCTTTGTGCCACAAGTTGCGGTGAC |  |  |
| Behind- ABB&BAA-F2 | TTACCGAGTTGAGCTCTCGC | 60 | 1155 |
| Behind- ABB&BAA-R2 | AAAACTGCAGGGTGAGGGAATAGTTCTTCTGTTG |  |  |
| Forward-ABA&BAB-F | CCGCTCGAGCAATGGACCCAGGAACCTCTCT | 60 | 699 |
| Forward- ABA&BAB-R | CGAAGGTGAGTGGGAAGGAGAGC |  |  |
| Behind- ABA&BAB-F | GCTCTCCTTCCCACTCACCTTCG | 60 | 714 |
| Behind- ABA&BAB-R | AAAACTGCAGGGTGAGGGAATAGTTCTTCTGTTG |  |  |
| Forward-Mut 1-F | CCGCTCGAGCAATGGACCCAGGAACCTCTCT | 60 | 222 |
| Forward-Mut 1-R | ACAAGTTGCGATGACCCCTCAG |  |  |
| Behind-Mut 1-F | CTGAGGGGTCATCGCAACTTGT | 60 | 1192 |
| Behind-Mut 1-R | AAAACTGCAGGGTGAGGGAATAGTTCTTCTGTTG |  |  |
| Forward-Mut 2-F | CCGCTCGAGCAATGGACCCAGGAACCTCTCT | 60 | 219 |
| Forward-Mut 2-R | AGTTGCCATGACCGCCCAGAATC |  |  |
| Behind-Mut 2-F | GATTCTGGGCGGTCATGGCAACT | 60 | 1196 |
| Behind-Mut 2-R | AAAACTGCAGGGTGAGGGAATAGTTCTTCTGTTG |  |  |
| Forward-Mut 3-F | CCGCTCGAGCAATGGACCCAGGAACCTCTCT | 60 | 221 |
| Forward-Mut 3-R | CAAGCTGCCATGACCGCCCAGAATCTTGGTCTGGTC |  |  |
| Behind-Mut 3-F | GACCAGACCAAGATTCTGGGCGGTCATGGCAGCTTG | 60 | 1207 |
| Behind-Mut 3-R | AAAACTGCAGGGTGAGGGAATAGTTCTTCTGTTG |  |  |
| Forward-Mut 4-F | CCGCTCGAGCAATGGACCCAGGAACCTCTCT | 60 | 230 |
| Forward-Mut 4-R | TTTGTGCCAGAAGCTGCCATGACCGCCCAGAATCTTGGTCTGGTTAGAAT |  |  |
| Behind-Mut 4-F | ATTCTAACCAGACCAAGATTCTGGGCGGTCATGGCAGCTTCTGGCACAAA | 60 | 1212 |
| Behind-Mut 4-R | AAAACTGCAGGGTGAGGGAATAGTTCTTCTGTTG |  |  |
| Forward-Mut 5-F | CCGCTCGAGCAATGGACCCAGGAACCTCTCT | 60 | 233 |
| Forward-Mut 5-R | GGCTGTGTGCCAGAAGCTGCCATGACCGCCCAGAATCTTGGTCTGGTTAGAATTTTTCCAATTGAAAGGT |  |  |
| Behind-Mut 5-F | ACCTTTCAATTGGAAAAATTCTAACCAGACCAAGATTCTGGGCGGTCATGGCAGCTTCTGGCACACAGCC | 60 | 1229 |
| Behind-Mut 5-R | AAAACTGCAGGGTGAGGGAATAGTTCTTCTGTTG |  |  |
| Forward-Mut 6-F | CCGCTCGAGCAATGGACCCAGGAACCTCTCT | 60 | 222 |
| Forward-Mut 6-R | AGAAGCTGCCGTGACTGCCCAG |  |  |
| Behind-Mut 6-F | CTGGGCAGTCACGGCAGCTTCT | 60 | 1192 |
| Behind-Mut 6-R | AAAACTGCAGGGTGAGGGAATAGTTCTTCTGTTG |  |  |
| Forward-Mut 7-F | CCGCTCGAGCAATGGACCCAGGAACCTCTCT | 60 | 228 |
| Forward-Mut 7-R | TGTGCCAGAAGCTGCGGTGACTCCTCAGAATCTTG |  |  |
| Behind-Mut 7-F | CAAGATTCTGAGGAGTCACCGCAGCTTCTGGCACA | 60 | 1199 |
| Behind-Mut 7-R | AAAACTGCAGGGTGAGGGAATAGTTCTTCTGTTG |  |  |
| Forward-Mut 8-F | CCGCTCGAGCAATGGACCCAGGAACCTCTCT | 60 | 219 |
| Forward-Mut 8-R | AGTTGCGGTGACTGCCCAGAATC |  |  |
| Behind-Mut 8-F | GATTCTGGGCAGTCACCGCAACT | 60 | 1196 |
| Behind-Mut 8-R | AAAACTGCAGGGTGAGGGAATAGTTCTTCTGTTG |  |  |
| Forward-Mut 9-F | CCGCTCGAGCAATGGACCCAGGAACCTCTCT | 60 | 219 |
| Forward-Mut 9-R | AGTTGCCGTGACTCCTCAGAATC |  |  |
| Behind-Mut 9-F | GATTCTGAGGAGTCACGGCAACT | 60 | 1196 |
| Behind-Mut 9-R | AAAACTGCAGGGTGAGGGAATAGTTCTTCTGTTG |  |  |
| Forward-Mut 10-F | CCGCTCGAGCAATGGACCCAGGAACCTCTCT | 60 | 219 |
| Forward-Mut 10-R | AGTTGCCGTGACTGCCCAGAATC |  |  |
| Behind-Mut 10-F | GATTCTGGGCAGTCACGGCAACT | 60 | 1196 |
| Behind-Mut 10-R | AAAACTGCAGGGTGAGGGAATAGTTCTTCTGTTG |  |  |
| Forward-Mut 11-F | CCGCTCGAGCAATGGACCCAGGAACCTCTCT | 60 | 219 |
| Forward-Mut 11-R | AGCTGCCATGACCCCTCAGAATC |  |  |
| Behind-Mut 11-F | GATTCTGAGGGGTCATGGCAGCT | 60 | 1196 |
| Behind-Mut 11-R | AAAACTGCAGGGTGAGGGAATAGTTCTTCTGTTG |  |  |
| Forward-Mut 12-F | CCGCTCGAGCAATGGACCCAGGAACCTCTCT | 60 | 219 |
| Forward-Mut 12-R | AGCTGCGATGACCGCCCAGAATC |  |  |
| Behind-Mut 12-F | GATTCTGGGCGGTCATCGCAGCT | 60 | 1196 |
| Behind-Mut 12-R | AAAACTGCAGGGTGAGGGAATAGTTCTTCTGTTG |  |  |
| Forward-Mut 13-F | CCGCTCGAGCAATGGACCCAGGAACCTCTCT | 60 | 219 |
| Forward-Mut 13-R | AGCTGCGATGACCCCTCAGAATC |  |  |
| Behind-Mut 13-F | GATTCTGAGGGGTCATCGCAGCT | 60 | 1196 |
| Behind-Mut 13-R | AAAACTGCAGGGTGAGGGAATAGTTCTTCTGTTG |  |  |
